# Differences in Soil Microbial Communities between Healthy and Diseased *Lycium barbarum* cv. Ningqi-5 Plants with Root Rot

**DOI:** 10.3390/microorganisms11030694

**Published:** 2023-03-08

**Authors:** Chenbo Jia, Yingrui An, Zhongyu Du, Huihui Gao, Jianyu Su, Chunyan Xu

**Affiliations:** 1 Key Laboratory of Ministry of Education for Protection and Utilization of Special Biological Resources, School of Life Sciences, Ningxia University, Yinchuan 750021, China; 2 Research Institute of Subtropical Forestry, Chinese Academy of Forestry, Hangzhou 311400, China

**Keywords:** *Lycium barbarum*, root rot, soil, microbial community structure, function prediction

## Abstract

For a long time, the development of the *Lycium barbarum* industry has been seriously restricted by root rot disease. In general, the occurrence of plant root rot is considered to be closely related to the composition and diversity of the soil microbial community. It is critical to understand the relationship between the occurrence of root rot in *L. barbarum* and the soil microbial composition. In this study, samples of the rhizosphere, rhizoplane, and root zone were collected from diseased and healthy plants. The V3–V4 region of bacterial 16S rDNA and the fungal ITS1 fragment of the collected samples were sequenced using Illumina MiSeq high-throughput sequencing technology. The sequencing results were first quality controlled and then aligned with the relevant databases for annotation and analysis. The richness of fungal communities in the rhizoplane and root zone of the healthy plants was significantly higher than that of the diseased plants (*p* < 0.05), and the community evenness and diversity of all the rhizoplane samples were significantly different from those of the rhizosphere and root zone. The richness of the bacterial communities in the rhizosphere and root zone of healthy plants was significantly greater than those of diseased plants (*p* < 0.05). The community composition of the rhizoplane was quite different from the other parts. The abundance of *Fusarium* in the rhizoplane and rhizosphere soil of diseased plants was higher than that in the corresponding parts of healthy plants. The abundances of *Mortierella* and *Ilyonectria* in the three parts of the healthy plants were correspondingly higher than those in the three parts of the diseased plants, and *Plectosphaerella* was the most abundant in the rhizoplane of diseased plants. There was little difference in the composition of the dominant bacteria at the phylum and genus levels between healthy plants and diseased plants, but the abundances of these dominant bacteria were different between healthy and diseased plants. Functional prediction showed that the bacterial community had the largest proportion of functional abundance belonging to metabolism. The functional abundances of the diseased plants, such as metabolism and genetic information processing, were lower than those of the healthy plants. The fungal community function prediction showed that the Animal Pathogen-Endophyte-Lichen Parasite-Plant Pathogen-Soil Saprotroph-Wood Saprotroph group had the largest functional abundance, and the corresponding fungi were *Fusarium*. In this study, we mainly discussed the differences in the soil microbial communities and their functions between the healthy and diseased *L. barbarum* cv. Ningqi-5, and predicted the functional composition of the microbial community, which is of great significance to understanding the root rot of *L. barbarum*.

## 1. Introduction

Many studies have illustrated the relationship between soil microorganisms and the occurrence of plant root rot. It has been confirmed that the occurrence of root rot is closely related to changes in soil microbial diversity and species composition, which has opened up new directions for research and has been applied to the study of root rot in various plants, such as American ginseng [1], *Aconitum carmichaelii* (Fuzi) [2], and *Coptis chinensis* [3]. The diversity of microorganisms in soil is extremely rich, comprising a large number of soil microbial species and functional groups. Soil microorganisms are one of the important regulators that maintain soil ecosystem functions and properties [4]. The diversity and composition of soil communities determine the multifunctionality of ecosystems [5]. Scientists have shown that the microbial community has a great impact on plants, including disease suppression [6] and adaptation to environmental changes [7]. Rhizosphere microorganisms mainly exist in a thin layer of soil around plant roots. Compared with the microorganisms in rhizosphere soil and root zone soil, rhizoplane microorganisms, which focus on root-surface-associated microbial communities, are a community that interacts closely with roots [8]. The rhizoplane is inhabited by a large number of different microbial communities [9], but its relationship with the occurrence of plant root rot is less studied. Second-generation high-throughput sequencing technology has been widely used in microbiome research, providing a powerful technical means for the analysis of microbial community species composition and species diversity [10]. It can more rapidly and efficiently clarify the structural composition of various microbial communities in complex samples [11] and help us to study the biological functions of microorganisms [12].

*Lycium barbarum*, a plant of the Solanaceae family, is an important medicinal and edible crop. Its fruit, leaves, and root bark have been used by people for a long time [13]. *L. barbarum* has the functions of nourishing the liver and kidneys and replenishing vital essence [14]. The polysaccharides and pigments of *L. barbarum* have shown great potential in anti-oxidation, lowering blood lipids, and improving immunity [15], occupying an important position in traditional Chinese medicine. Ningxia, Gansu, Qinghai, Xinjiang, Inner Mongolia, and Hebei are the main production areas for Chinese wolfberry [16], among which *L. barbarum* from Ningxia is usually considered to possess the highest quality [17] and is mainly exported [18] to Asian and European markets. The planting area of *L. barbarum* in Ningxia accounts for nearly half of the country’s planting area. With the continuous expansion of the planting scale of *L. barbarum* in Ningxia, *L. barbarum* diseases have been paid more attention to. Root rot [19] is one of the common diseases of *L. barbarum* and often leads to a reduction in fruit yield, resulting in a serious problem for the *L. barbarum* industry. At present, there are many studies on root rot involving soybean [20,21], ginseng [22], and other crops, while few studies on *L. barbarum* root rot have been reported. These mostly focus on the occurrence status of *L. barbarum* root rot and preventive measures. Since the beginning of the twentieth century, researchers have successively found the pathogens of root rot on apples [23], *Pinus radiata* [24], and other plants. These are mainly *Pythium*, *Phytophthora*, *Rhizoctonia solani* [25], *Fusarium*, *Sclerotinia sclerotiorum,* and other common pathogens that cause soil-borne diseases of plants.

A survey conducted in Zhongning County, Ningxia, found that after root rot of *L. barbarum* occurs, the roots and rhizomes have different degrees of rot, severely exposed xylem, and the visible vascular bundles are dark brown. At present, the pathogens found in *L. barbarum* in Ningxia include *Fusarium oxysporum*, *Fusarium solani*, *Fusarium concolor*, *Fusarium moniliforme*, *Fusarium equiseti*, *Fusarium incarnatum,* and *Rhizoctonia solani*. They affect not only the growth of *L. barbarum* plants but also the formation of fruits. If the roots of a plant have ponding water or the plant is in a relatively humid environment, a white or pink mold layer will appear in the surrounding soil and diseased parts, and it is necessary to more systematically and deeply understand the differences in the soil microbial community structure between the healthy and the diseased plants of *L. barbarum* in Ningxia. In this study, the root tissues, rhizosphere, and root zone soil of healthy and diseased *L. barbarum* cv. Ningqi-5 plants were first collected, then processed to obtain rhizoplane, rhizosphere, and root zone samples for high-throughput sequencing in order to compare the diversity and community structure of microorganisms found in different areas of the diseased and healthy plants. This research will help to clarify the relationship between *L. barbarum* root rot and the microbial community and provide a theoretical reference for the biological control of *L. barbarum* root rot in the future. In this study, we aim to elucidate the differences in soil microbial community structure between diseased and healthy *L. barbarum* plants and identify key pathogens.

## 2. Materials and Methods

### 2.1. Location Description

**Figure 1:**
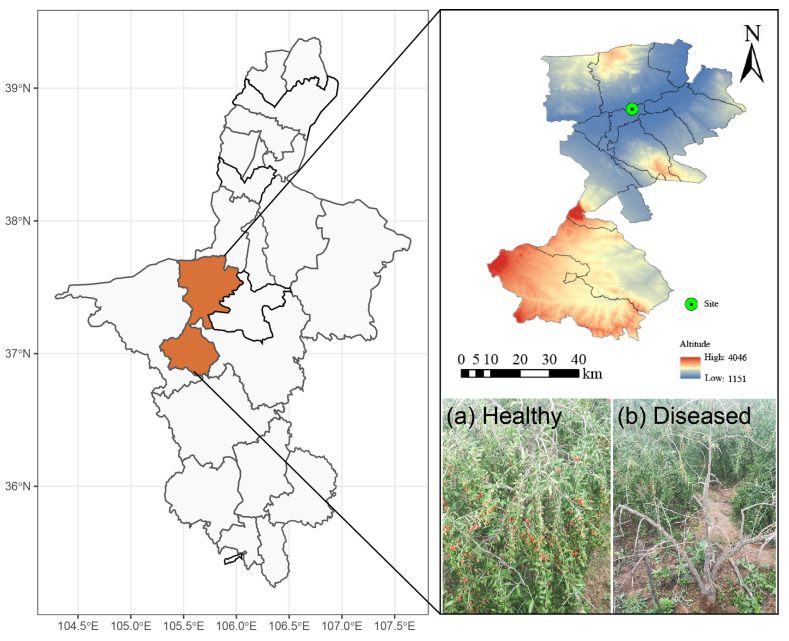
Diagram of the sampling site. Note: The upper figure shows the sampling site, and the lower-right part of the figure shows a healthy Chinese wolfberry plant and a Chinese wolfberry plant with root rot disease.

The sampling site is located at Ningxia QiXin Wolfberry Seedlings Cooperative in Zhongning County, Ningxia, China (37°32′ N, 105°43′ E; Figure 1), which has a temperate continental monsoon climate and long-day photoperiod. The mean annual temperature is 8.3–11.0 °C, and the mean annual precipitation is about 300 mm. The terrain is high in the south and low in the north, with an average altitude of 1140–1600 m and average annual precipitation of 78.5–335.0 mm. The terrain of Zhongning County is flat, and the soil layer is deep. Due to the confluence of the Yellow River and the Qingshui River, the soil is rich in various minerals and trace elements, which provides better soil conditions for the cultivation of *L. barbarum*.

### 2.2. Sample Collection

First, possibly diseased plants were selected based on the morphology of their stems and leaves. Then, the roots of the selected *L. barbarum* plants were dug out to further judge whether the plant suffered from root rot disease based on the root morphology. Three diseased plants suffering from root rot and 3 healthy *L. barbarum* plants were selected to collect samples. All the diseased plants and the healthy plants were selected from the same plantation. The diseased plants were selected according to the disease condition, and the healthy *L. barbarum* plants were randomly selected, generally within 10 m of the diseased plants.

Samples were collected from 3 directions of the diseased root (we divided the plant into 3 pieces, sampled it separately, and then mixed it together after taking it.), the root zone, rhizosphere soil and roots were collected in turn based on the principle of “from far to near” to the root of the corresponding samples. We mixed the root tissues collected from 3 different directions to create sample QX111. Two other diseased samples, QX112 and QX113, were collected in a similar fashion. The average value of these 3 samples (QX111, QX112 and QX113), collectively named QX_GBBZ, was used for the analysis of microorganisms in the rhizoplane of the diseased plants. Similarly, 3 soil samples (QX121, QX122, and QX123) from the rhizosphere of the diseased plants were collected and named QX_GJBZ using the average value during analysis. The root zone samples of the diseased plants were denoted QX131, QX132, and QX133, and the 3 samples were combined and named QX_GWBZ during analysis. The 3 samples (QX114, QX115, and QX116) of the rhizoplane of the healthy plants were collected using the same method, and the 3 samples were combined and named QX_GBJK during analysis. The rhizosphere samples of the healthy plants were named QX124, QX125, and QX126, respectively, and the 3 samples were collectively named QX_GJJK. The root zone samples of the healthy plants were denoted QX134, QX135, and QX136 and collectively named QX_GWJK during analysis.

The soil adhering to the roots was carefully removed with a soft brush and represented the rhizosphere soil, with about 20 g per sample. The soil sampled from a circle 1 cm away from the root was collected as the root zone soil sample, with about 100 g per sample. The root tissues were brought back to the laboratory for processing to obtain the rhizoplane samples. The specific sample numbers are shown in Table 1.

**Figure 2:**
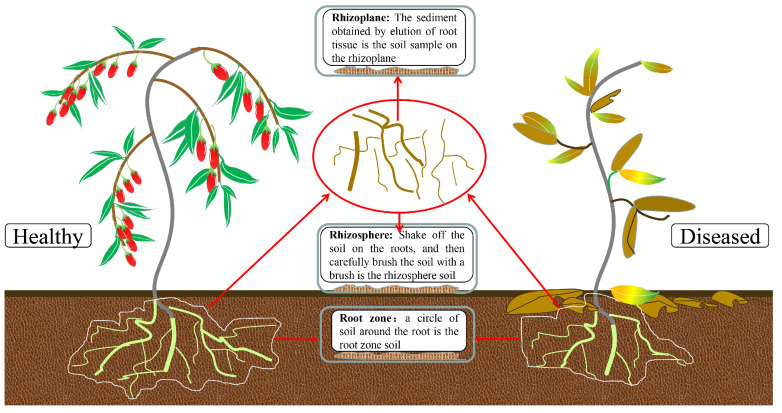
Diagram of sample collection.

The collected samples were packed and numbered using sterile plastic bags, sealed, and brought back to the laboratory for refrigeration at −20 °C. Root tissue was collected for the rhizoplane samples, and soil samples were collected from the rhizosphere and root zone. In addition, all the selected healthy and diseased *L. barbarum* plants were 5 years old. After collection, all the samples were immediately placed in a low-temperature box filled with ice packs and quickly refrigerated at −20 °C. The sampling diagram is shown in Figure 2.

### 2.3. Sample Processing

The rhizosphere and root zone soil samples were temporarily taken out of refrigeration, then quickly sieved through a 20-mesh standard sieve to remove stones and debris and used for the extraction of total soil DNA.

The root tissues were immersed in phosphate buffer solution and incubated twice at 4 °C at 180 r·min^−1^ for 20 min. The washing solution was collected and centrifuged at 10,000 r·min^−1^ for 10 min at 4 °C [8]. The precipitate was collected to obtain the rhizoplane samples and numbered the same as the name corresponding to the root tissue.

### 2.4. DNA Extraction and PCR Amplification

According to the manufacturer’s instructions, the metagenomic DNA of each microbial community was extracted using a DNeasy PowerSoil Pro Kit (QIAGEN’s Inhibitor Removal Technology^®^ Second-generation). The concentration of the DNA extracts was checked using 1% agarose gel, and the purity of the DNA samples was determined with a NanoDrop 2000 UV-vis spectrophotometer (Thermo Scientific, Wilmington, NC, USA). The hypervariable regions V3-V4 of the bacterial 16S rRNA gene were amplified with primer pairs 338F (5’-ACTCCTACGGGAGGCAGCAG- 3’) and 806R (5’-GGACTACHVGGGTWTCTAAT-3’) using an ABI GeneAmp ^®^ 9700 PCR thermocycler (ABI, CA, USA). The ITS1 regions of the fungi were amplified with primer pairs ITS1F (5’-CTTGGTCATTTAGAGGAAGTAA-3’) and ITS2R (5’-GCTGCGTTCTTCATCGATGC-3’). The PCR amplification was performed as follows: initial denaturation at 95 °C for 3 min followed by 35 cycles of denaturing at 95 °C for 30 s, annealing at 55 °C for 30 s, extension at 72 °C for 45 s, single extension at 72 °C for 10 min, and ending at 10 °C. The PCR mixtures contained 5 × TransStart FastPfu buffer 4 μL, 2.5 mM dNTPs 2 μL, forward primer (5 μM) 0.8 μL, reverse primer (5 μM) 0.8 μL, TransStart FastPfu DNA Polymerase 0.4 μL, template DNA 10 ng and finally ddH2O up to 20 μL. PCR reactions were performed in triplicate. The PCR product was extracted from 2% agarose gel and purified using an AxyPrep DNA Gel Extraction Kit (Axygen Biosciences, Union City, CA, USA) according to the manufacturer’s instructions and quantified using a Quantus™ Fluorometer (Promega, Madison, WI, USA).

### 2.5. Illumina MiSeq Sequencing

Using Majorbio Bio-Pharm Technology Co. Ltd. (Shanghai, China)’s standard protocols, purified amplicons were pooled in equimolar concentrations and then paired-end sequenced (2 × 300) on an Illumina MiSeq platform (Illumina, San Diego, CA, USA).

After being demultiplexed according to fastp version 0.20.0 [26] and merged using FLASH version 1.2.7 [27], the raw 16S rRNA gene sequencing reads were quality-filtered with the following criteria: (i) The 300 bp reads were truncated at any site receiving an average quality score of <20 over a 50 bp sliding window, and the truncated reads shorter than 50 bp and reads containing ambiguous characters, were discarded. (ii) Only overlapping sequences longer than 10 bp were assembled according to their overlapped sequence. The maximum mismatch ratio of the overlap region was 0.2. (iii) Samples were distinguished according to their barcode and primers, and the sequence direction was adjusted with exact barcode matching and 2-nucleotide mismatching in respect of primer matching.

### 2.6. Statistical Analysis

We clustered operational taxonomic units (OTUs) based on a 97% similarity cutoff using UPARSE version 7.1 [28] and removed chimeric sequences. RDP Classifier Version 2.2 [29] was used to analyze the taxonomy of each OTU representative sequence against the 16S rRNA database using a confidence threshold of 0.7. Excel 2016 and SPSS 17.0 were used for data statistics and analysis. A 1-way ANOVA was used to test significance, followed by a Duncan test (*p* < 0.05), and all data were expressed as “mean ± standard deviation.”

## 3. Results

### 3.1. Microbial Alpha Diversity of Soil of Ningqi-5

**Figure 3:**
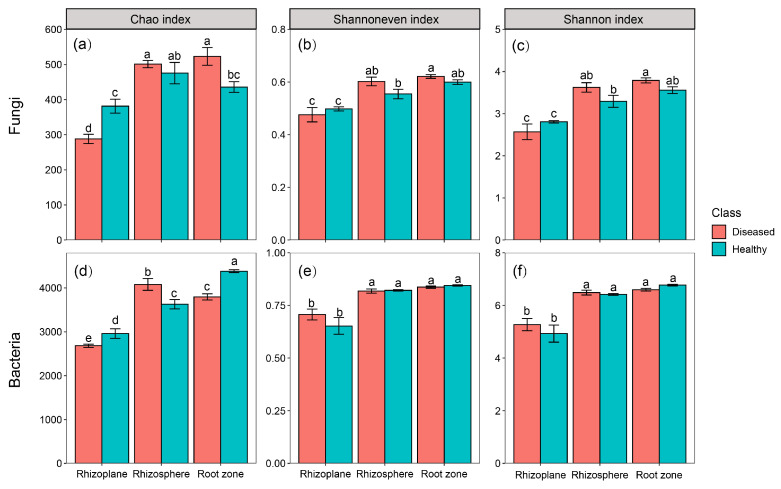
Alpha diversity indexes of the microbial communities. Note: Different letters indicate significant differences (*p*  <  0.05). Data are presented as mean ± standard deviation. (**a**) denotes the chao index of the fungal community, (**b**) denotes the shannoneven index of the fungal community, (**c**) denotes the shannon index of the fungal community, (**d**) denotes the chao index of the bacterial community, (**e**) denotes the shannoneven index of the bacterial community, (**f**) denotes the shannon index of the bacterial community.

After sequencing all samples, we found there were 1,036,026 effective fungal sequences generated, clustered into 1463 OTUs, and 435,420 effective bacterial sequences generated, clustered into 6783 OTUs. The microbial community coverage of the rhizoplane, rhizosphere, and root zone soil was each higher than 0.95 (Figure 3), indicating that the sequencing depth of the samples generally reflected the real situation in the microbial community.

The fungal community richness in the rhizoplane of the healthy plants was significantly greater than that in the rhizoplane of the diseased plants (*p* < 0.05). The community richness of the healthy plants and the diseased plants in the root zone was also significantly different (*p* < 0.05). In all the healthy and diseased plants, the evenness and diversity of the community in the rhizoplane were significantly different from those of the rhizosphere and root zone (*p* < 0.05). For the bacterial community, the richness of the healthy plants was greater than that of the diseased plants, but there was no significant difference in the rhizoplane (*p* > 0.05). There was no significant difference in community richness, evenness, and diversity between the healthy and the diseased plants.

### 3.2. Principal Coordinate Analysis Based on OTU Level

**Figure 4:**
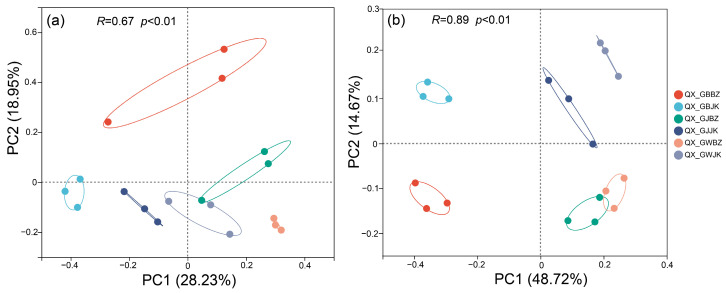
PCoA analysis at OTU level. Note: The PC coordinate axis in the principal coordinate analysis (PCoA) graph denotes the explanatory value of the difference in sample composition. (**a**) denotes the fungal community, and (**b**) denotes the bacterial community. QX_GBBZ denotes the rhizoplane diseased plants, QX_GBJK denotes the rhizoplane healthy plants, QX_GJBZ denotes the rhizosphere diseased plants, QX_GJJK denotes the rhizosphere healthy plants, QX_GWBZ denotes the root zone diseased plants, and QX_GWJK denotes the root zone healthy plants.

The principal coordinate analysis (PCoA) based on the Bray–Curtis distance detected a total variation of 47.18% in the fungal communities (Figure 4a). In the fungal communities, it can be clearly shown that the rhizoplane samples of the diseased plants were distinguished from other samples by PC1. The rhizosphere of the healthy plants tended to be close to the rhizoplane of the diseased plants, which indicates that the community composition of the two groups was different. The total variation between bacterial communities was 63.39% (Figure 4b), in which samples of the healthy plants and the diseased plants were clearly distinguished by PC1, which means samples of the healthy and the diseased plants corresponding to the three parts were significantly different in their community composition. In addition, the rhizoplane samples of the healthy and diseased plants were also distinguished by PC2, suggesting that there were great differences in the composition of community structure between the rhizoplane and the other two parts.

### 3.3. Analysis of Microbial Community Composition

**Figure 5:**
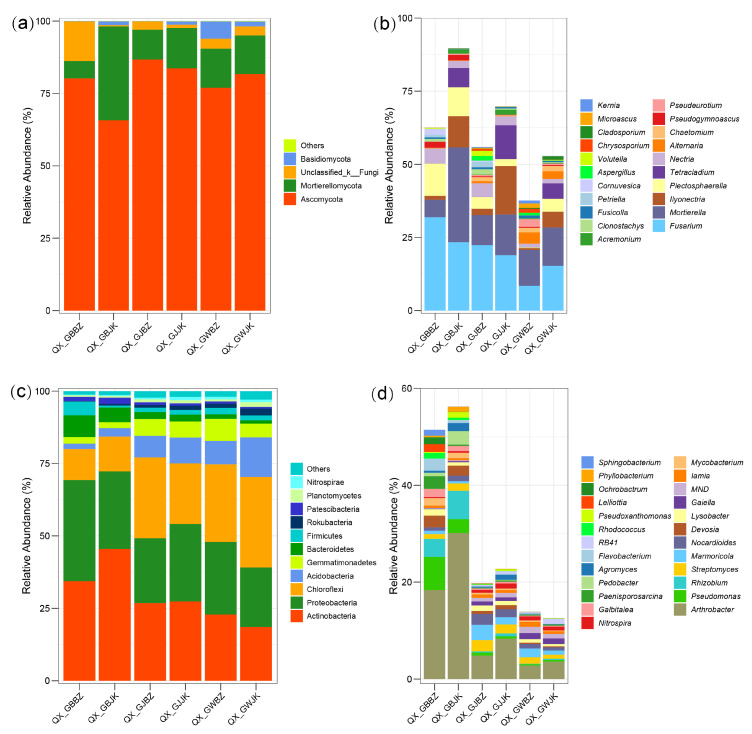
Relative abundance of fungal and bacterial communities at the phylum and genus levels. Note: (**a**) denotes the phyla of fungi, (**b**) denotes the genera of fungi, (**c**) denotes the phyla of bacteria, and (**d**) denotes the genera of bacteria. QX_GBBZ denotes the rhizoplane diseased plants, QX_GBJK denotes the rhizoplane healthy plants, QX_GJBZ denotes the rhizosphere diseased plants, QX_GJJK denotes the rhizosphere healthy plants, QX_GWBZ denotes the root zone diseased plants, and QX_GWJK denotes the root zone healthy plants.

At the phylum level, Ascomycota was the dominant phylum in all the samples, accounting for an average abundance of 79.14%, with the highest abundance in the rhizosphere of the diseased plants (86.73%). Mortierellomycota were the next most abundant fungi, with an average proportion of 14.92%. There was a significant difference in the abundance of Mortierellomycota in rhizoplane soil between the healthy and diseased plants. Specifically, the proportion of relative abundance of Mortierellomycota in the healthy plants’ rhizoplane soil was 32.43%, and in the diseased plants’ rhizoplane soil, it was 5.94% (Figure 5A).

At the genus level (Figure 5B), the definite species with abundance exceeding 1% in each sample accounted for 37.64%–89.67%. The main fungi in the root zone soil of the healthy plants were *Fusarium* (15.33%), *Mortierella* (13.13%), *Ilyonectria* (5.33%), *Tetracladium* (5.28%), and *Plectosphaerella* (4.42%). The main fungi in the root zone soil of the diseased plants were *Mortierella* (12.26%), *Fusarium* (8.50%), *Alternaria* (3.84%), *Pseudeurotium* (2.64%), and *Chaetomium* (1.60%). It can be seen that the composition and abundance of dominant genera in the root zone soil of the healthy and diseased plants were quite different. The abundances of *Fusarium* and *Mortierella* in the root zone soil of the diseased plants were, respectively, 44.55% and 6.63% lower than in the root zone soil of the healthy plants. The main fungi in the rhizosphere soil of the healthy plants were *Fusarium* (18.94%), *Ilyonectria* (16.49%), *Mortierella* (13.97%), *Tetracladium* (11.63%), and *Nectria* (2.96%). The main fungi in the rhizosphere soil of the diseased plants were *Fusarium* (22.37%), *Mortierella* (10.28%), *Nectria* (4.66%), *Plectosphaerella* (4.03%), and *Ilyonectria* (2.16%). In the rhizosphere soil, there were no significant differences between healthy and diseased plants in terms of dominant genera. However, there was a significant difference in the abundance of dominant genera. The abundances of *Fusarium* and *Nectria* in the rhizosphere soil of diseased plants were, respectively, 18.11% and 57.43% higher than in the rhizosphere soil of the healthy plants, while the abundances of *Ilyonectria* and *Mortierella* were, respectively, 86.90% and 26.41% lower in the rhizosphere soil of diseased plants versus that of healthy plants. The dominant fungi in the rhizoplane of the healthy plants were *Mortierella* (32.42%), *Fusarium* (23.39%), *Ilyonectria* (10.69%), *Plectosphaerella* (9.81%), and *Tetracladium* (6.59%). The dominant fungi in the rhizoplane of the diseased plants were *Fusarium* (31.95%), *Plectosphaerella* (11.06%), *Mortierella* (5.94%), *Nectria* (5.12%), and *Cornuvesica* (2.09%). It can be seen that the composition of dominant genera in the rhizoplane of the healthy and diseased plants had no notable differences, but there was a great difference in abundance. The abundance of *Mortierella* in the rhizoplane soil of the diseased plants was 81.68% lower than that in the rhizoplane soil of healthy plants, while the abundances of *Fusarium* and *Plectosphaerella* were 36.59% and 12.74% higher, respectively. In addition, *Plectosphaerella* was most abundant in the rhizoplane of the diseased plants.

The dominant fungi and their proportions in the three parts of the healthy and diseased plants were quite different. The abundance of the dominant genus *Fusarium* in the rhizoplane and rhizosphere soil of the diseased plants was higher than that in the corresponding parts of the healthy plants, while the abundances of *Mortierella* and *Ilyonectria* in the three parts of the healthy plants were higher than those in the three corresponding parts of the diseased plants.

In the bacterial community, Actinobacteria, Proteobacteria, Chloroflexi, Acidobacteria, and Gemmatimonadetes were the dominant phyla (Figure 5c). The abundances of Actinobacteria and Proteobacteria in the rhizoplane were greater than those in the rhizosphere and root zone, while the abundances of Chloroflexi, Acidobacteria, and Gemmatimonadetes in the rhizoplane were less than those in the rhizosphere and root zone.

At the genus level (Figure 5d), the definite species with an abundance higher than 1% in each sample accounted for 12.58%-56.26%. The main bacteria in the root zone soil of the healthy plants were *Arthrobacter* (3.6%), *Gaiella* (1.18%), RB41 (1.07%), MND1 (0.90%), and *Marmoricola* (0.85%), and the main bacteria in the root zone soil of the diseased plants were *Arthrobacter* (2.78%), *Marmoricola* (1.83%), *Streptomyces* (1.33%), MND1 (1.29%), and *Gaiella* (1.26%). It can be seen that there were no significant differences in the composition and abundance of dominant genera between the healthy and diseased plants in the root zone. The main bacteria in the rhizosphere soil of the healthy plants were *Arthrobacter* (8.34%), *Streptomyces* (1.81%), *Nocardioides* (1.66%), *Marmoricola* (1.50%), and *Nitrospira* (1.06%), and the main bacteria in the rhizosphere soil of the diseased plants were *Arthrobacter* (4.83%), *Marmoricola* (3.12%), *Streptomyces* (2.36%), *Nocardioides* (2.24%), and *Lysobacter* (1.14%). It can be seen that the composition of dominant genera in the rhizosphere soil of the healthy and diseased plants had no significant difference, but there was a certain difference in abundance. The abundance of *Arthrobacter* in the rhizosphere soil of the diseased plants was 42.09% lower than in the rhizosphere soil of the healthy plants, and the abundances of *Nocardioides*, *Marmoricola,* and *Streptomyces* in the rhizosphere soil of the diseased plants were 25.89%, 51.92%, and 23.31% higher than those in the rhizosphere soil of the healthy plants, respectively. The main bacteria in the rhizoplane soil of the healthy plants were *Arthrobacter* (30.14%), *Rhizobium* (5.88%), *Pseudomonas* (2.84%), *Pedobacter* (2.78%), and *Devosia* (2.06%), and the main bacteria in the rhizoplane soil of the diseased plants were *Arthrobacter* (18.39%), *Pseudomonas* (6.83%), *Rhizobium* (3.70%), *Paenisporosarcina* (2.62%), and *Devosia* (2.45%). It can be seen that in the rhizoplane, the healthy and the diseased plants had no notable differences in the composition of dominant genera, but there was a certain difference in abundance. In particular, the abundance of *Arthrobacter* and *Pseudomonas* changed greatly. The abundance of *Arthrobacter* in the rhizoplane soil of the diseased plants was 38.98% lower than that seen in the healthy plants, and the abundance of *Pseudomonas* in the rhizoplane soil of the diseased plants was 140% higher than that seen in the healthy plants.

There were no major differences in the composition of dominant bacteria in the three parts of the healthy and diseased plants, but there were certain differences in abundance. The abundances of *Arthrobacter* and *Pseudomonas* in the rhizoplane differed greatly between the healthy and diseased plants, and the abundances of *Arthrobacter* and *Pseudomonas* were much greater than in the rhizosphere and root zone.

### 3.4. Significant Difference between Each Group

**Figure 6:**
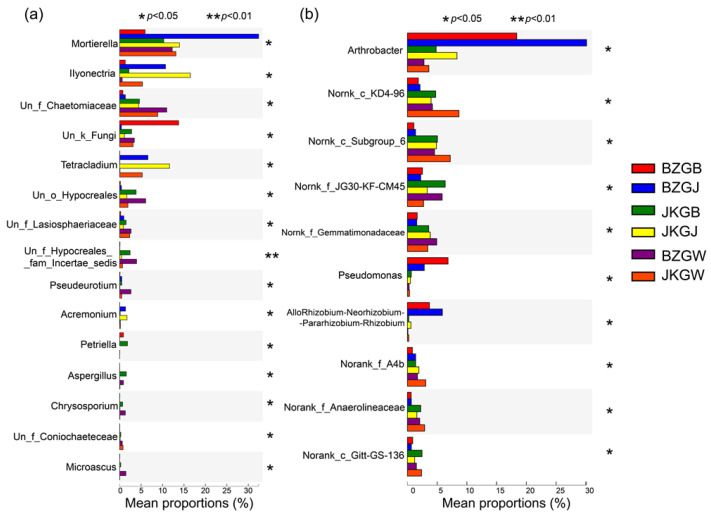
Kruskal–Wallis H test bar plot. Note: The Y-axis represents the species names at the genus level. The X-axis represents the average relative abundance in different groups, and the columns with different colors represent different groups. On the far right is the *p*-value, and “*” represents 0.01 < *p* ≤ 0.05. (**a**) Is the fungal community, and (**b**) is the bacterial community. BZGB denotes the rhizoplane diseased plants, JKGB denotes the rhizoplane healthy plants, BZGJ denotes the rhizosphere diseased plants, JKGJ denotes the rhizosphere healthy plants, BZGW denotes the root zone diseased plants, and JKGW denotes the root zone healthy plants.

Based on the community abundance data, the Kruskal–Wallis H test, which can be used to carry out significant difference analysis of species in multiple groups of samples, was used to test the hypothesis and evaluate the significance of the observed differences. With multiple test correction methods on the P value with “fdr (Falsely Discovery Rate),” the top ten species in terms of relative abundance were screened. After removing unclear species, such as no-rank and unclassified groups, the species observed in the fungal community were *Fusarium*, *Mortierella*, *Ilyonectria*, *Plectosphaerella*, *Tetracladium,* and *Nectria*. Among these, the species with significant differences in each sample were *Mortierella*, *Ilyonectria,* and *Tetracladium*, and their abundance in the healthy plants was significantly higher than in the diseased plants (Figure 6A). Similarly, after removing the unclear species, such as no-rank and unclassified groups in the top ten species of relative abundance, the species observed in the bacterial community were *Arthrobacter*, *Pseudomonas,* and *Rhizobium*, and there were significant differences in each sample (Figure 6B). The abundances of Arthrobacter, *Pseudomonas,* and *Rhizobium* in the rhizoplane were much greater than those in the rhizosphere and root zone. The abundances of *Arthrobacter* and *Rhizobium* in the three parts of the healthy plants were significantly greater than those in the diseased plants.

### 3.5. Prediction of Microbial Function

**Figure 7:**
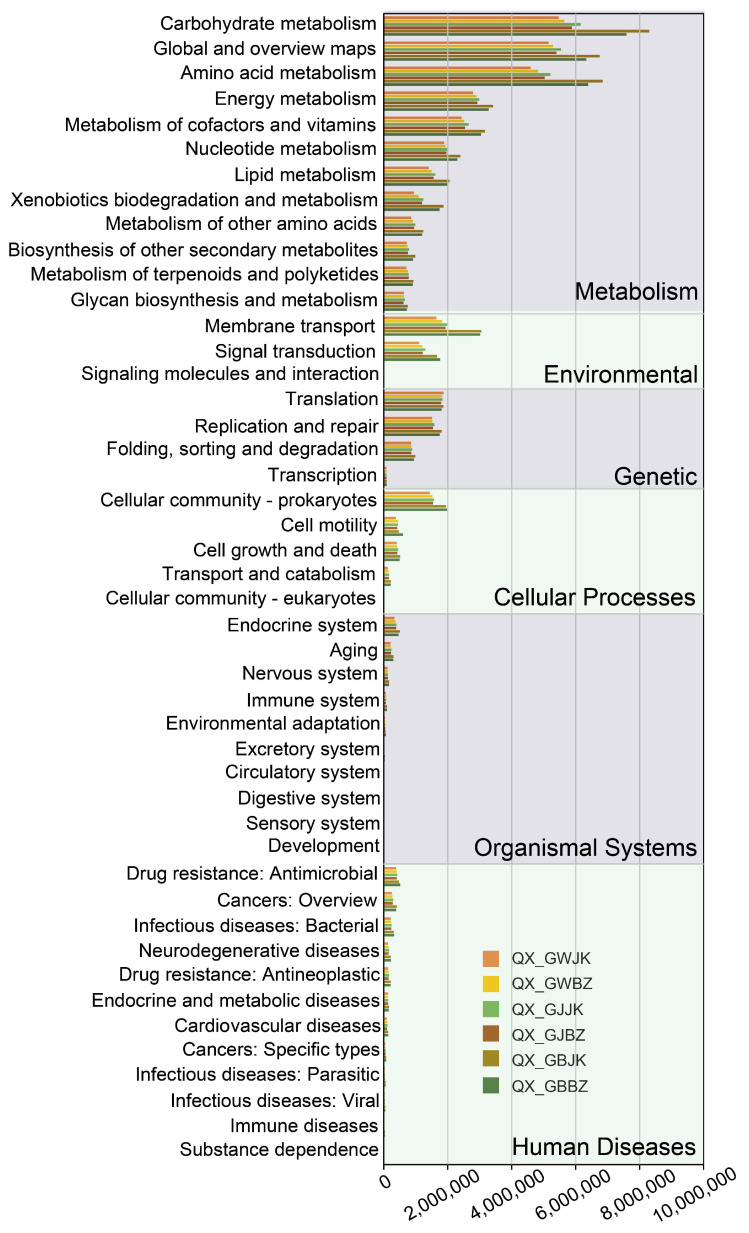
Functional prediction of the bacterial community. Note: QX_GBBZ denotes the rhizoplane diseased plants, QX_GBJK denotes the rhizoplane healthy plants, QX_GJBZ denotes the rhizosphere diseased plants, QX_GJJK denotes the rhizosphere healthy plants, QX_GWBZ denotes the root zone diseased plants, and QX_GWJK denotes the root zone healthy plants.

**Figure 8:**
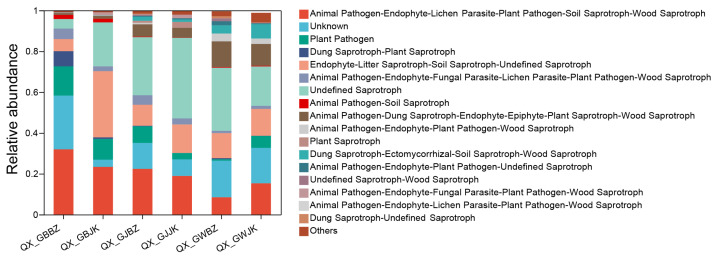
Prediction of the functional composition of the fungal community. Note: The abscissas from left to right represent rhizoplane diseased plant, rhizoplane healthy plant, rhizosphere diseased plant, rhizosphere healthy plant, root zone diseased plant, and root zone healthy plant. QX_GBBZ denotes the rhizoplane diseased plants, QX_GBJK denotes the rhizoplane healthy plants, QX_GJBZ denotes the rhizosphere diseased plants, QX_GJJK denotes the rhizosphere healthy plants, QX_GWBZ denotes the root zone diseased plants, and QX_GWJK denotes the root zone healthy plants.

In order to explore the differences in the functional distribution of the microbial communities in the diseased and healthy plants, PICRUSt2 and FUNGuild were used to predict the bacterial and fungal community function, respectively (Figure 7 and Figure 8), and the prediction results were compared with the KEGG database. It was found that the predicted functions of the bacterial community involved metabolism, environmental information processing, genetic information processing, cellular processes, organismal systems, and human diseases (Figure 7). Among them, the functional abundance involving metabolism accounted for the largest proportion, mainly including carbohydrate metabolism, global and overview maps, amino acid metabolism, energy metabolism, metabolism of cofactors and vitamins, nucleotide metabolism, lipid metabolism, xenobiotics biodegradation and metabolism, metabolism of other amino metabolism acids, biosynthesis of other secondary metabolites, metabolism of terpenoids and polyketides, and glycan biosynthesis and metabolism. The function of environmental information processing mainly included membrane transport, signal transduction, signaling molecules, and interaction. The functional abundances of the rhizoplane of the diseased and healthy plants were greater than those of the rhizosphere and root zone, which may be related to the fact that the microorganisms in the rhizoplane were close to the plant, leading to greater interaction between the microorganisms and plants. Another possible explanation is that the rhizoplane is a more specialized ecological niche that demands the genetic machinery of the inhabitants (for example, the ability to attach to root surfaces and establish aggregates of biofilm structures, as well as avoiding the plant response machinery). In addition, the functional abundance of the rhizoplane of the healthy plants was greater than that of the diseased plants except for the root zone, indicating that the functions of metabolism and genetic information processing decreased to a certain extent due to disease.

According to the function prediction of FUNguild, the functional classification of fungi and the abundance information of each functional classification in different samples can be obtained (Figure 8). According to the way fungi obtain nutrients, fungi can be divided into three categories: pathotrophs, saprotrophs, and symbiotrophs. There was a relatively high functional abundance for the three groups comprising Animal Pathogen-Endophyte-Lichen Parasite-Plant Pathogen-Soil Saprotroph-Wood Saprotroph, Plant Pathogen, and Animal Pathogen-Endophyte-Fungal Parasite-Lichen Parasite-Plant Pathogen-Wood Saprotroph. Fungi in the Animal Pathogen-Endophyte-Lichen Parasite-Plant Pathogen-Soil Saprotroph-Wood Saprotroph group were all *Fusarium*, and the fungi corresponding to plant pathogens were relatively increased, including *Volutella*, *Clonostachys*, *Plectosphaerella*, *Cornuvesica*, *Leptosphaeria*, *Gibberella*, *Mycosphaerella*, *Lectera*, etc. It was *Acremonium* that corresponded to the Animal Pathogen-Endophyte-Fungal Parasite-Lichen Parasite-Plant Pathogen-Wood Saprotroph group. There were more functional abundances in the Animal Pathogen-Endophyte-Lichen Parasite-Plant Pathogen-Soil Saprotroph-Wood Saprotroph, and Plant Pathogen groups in the rhizosphere and rhizoplane of healthy plants than in those of diseased plants and the opposite was true in the Animal Pathogen-Endophyte-Fungal Parasite-Lichen Parasite-Plant Pathogen-Wood Saprotroph group.

## 4. Discussion

The fungal community richness in the rhizoplane of the healthy plants was significantly greater than that seen in the diseased plants, which is consistent with the changes in the fungal diversity in the soil of *Panax notoginseng* caused by root rot [30]. However, the α-diversity of soil microorganisms in the rhizosphere and root zone of the healthy and diseased plants did not show large differences. In addition, the community evenness and diversity in the rhizoplane of the healthy and diseased plants were significantly different from those in the rhizosphere and root zone, which may be because the microorganisms in the rhizoplane were closer to the root system of the plants, and the secretions of plants caused the microbial community to change [31]. The closer the soil around the roots, the more compounds it contains, such as amino acids, organic acids, vitamins, polysaccharides, enzymes, etc. [32,33]. In the bacterial community, there was no significant difference in the α diversity of the healthy and diseased plants in the rhizosphere and root zone. It is also possible that because the rhizoplane soil microorganisms are closer to the plant roots, they are more affected by root exudates [34]. In the soil environment, pathogens are often affected by root exudates of host plants, leading to the occurrence of plant diseases and insect pests [35]. In addition, we found the lowest microbial α-diversity in the rhizoplane. This may be a result of root exudates capable of attracting beneficial microbial taxa, chelating toxic compounds in the soil, or changing soil properties, and suggests that plants employ positive strategies to “recruit” beneficial soil microbes to protect the distal part of the plant [36,37,38]. Therefore, the community richness of microorganisms in the rhizoplane is generally lower than that in the rhizosphere and root zone. PCoA analysis also clearly showed that the rhizoplane of the diseased plants had the greatest difference in community composition compared with other samples. In addition, there were some differences in the community composition of each sample from the healthy and the diseased plants.

The dominant fungi and their proportions in the three parts of the healthy and diseased plants were quite different, indicating that the composition and abundance of the dominant fungi were in a state of dynamic change from the root zone to the rhizosphere and the rhizoplane. This may be caused by root exudates, which have been shown to be key mediators in the interaction between plants and soil microbial communities [39]. These substances form a unique living microenvironment for the microorganisms of plants’ roots, and different microorganisms have different responses to the compounds released by the plant roots [40]. The complex and diverse plants and their associated microbial communities are the keys to understanding soil microecology [41,42]. However, little is known about the interactions between microbes in the community environment and the mechanisms that affect the function of plant-associated microbial communities. In this study, the dominant soil fungi were Basidiomycota and Ascomycota, but their relative abundances were quite different among the six groups of samples. Basidiomycota and Ascomycota are mostly saprophytes, which are fundamental decomposers in soil and play a key role in the degradation of complex organic matter [43]. *Fusarium*, the root rot pathogen of various crops such as peas [44] and soybeans [45], is a type of Ascomycota. The abundance of *Fusarium* in the rhizoplane and rhizosphere soil of the diseased plants was higher than that in the corresponding parts of the healthy plants, and the abundance of *Fusarium* showed a downward trend from the rhizoplane to the rhizosphere and then to the root zone. The abundance of *Mortierella* in the three parts of the healthy plants was higher than that of the three corresponding parts of the diseased plants. Consistent with our expectations, it is speculated that *Fusarium* may be one of the important pathogens of *L. barbarum* root rot in Ningxia. However, the relationship between microorganisms and plants is complex. Therefore, in order to determine whether *Fusarium* is the key pathogen of *L. barbarum* root rot, it is necessary to isolate *Fusarium* from *L. barbarum* root through experiments and verify its pathogenicity through Koch’s rule. Studies have shown that *Mortierella* is a kind of relatively common beneficial bacteria. *Mortierella* can produce a variety of biologically active substances, which are closely related to soil fertility and can effectively promote plant growth and soil health [46]. It may also have biocontrol functions. In addition, *Plectosphaerella* was the most abundant in the rhizoplane of diseased plants, which caught our attention. *Plectosphaerella*, as a plant pathogen, was first reported in *Panax notoginseng* [47]. Recently, some researchers also reported that potato wilt is caused by *Plectosphaerella* [48], but whether it can cause root rot of *L. barbarum* deserves further study. The dominant phyla in the bacterial community were Proteobacteria, Actinobacteria, Chloroflexi, and Acidobacteria. There was no significant difference in the composition of dominant bacteria in the three parts of healthy plants and diseased plants, but there were certain differences in abundance. The abundances of *Arthrobacter* and *Pseudomonas* in the rhizoplane were significantly different between the healthy and the diseased plants. In addition, the abundances of *Arthrobacter* and *Pseudomonas* were much greater than in the rhizosphere and root zone. *Arthrobacter*, one typical example of the dominant genus in all the samples, were the more common bacteria in the soil, and are generally considered to be beneficial bacteria. Bacteria have evolved complex strategies, such as chelating iron by secreting siderophores, which subsequently alter the growth of nearby microorganisms [49]. Studies have confirmed that the acquisition and competition of bacteria for resources is one of the important factors in bacterial community composition and pathogen invasion in the tomato rhizosphere [50]. These results not only explain the impact of resource competition on microbial interaction, but also show their correlation with the health of plants.

*L. barbarum* is a perennial crop, and its soil is generally monoculture soil, which is extremely prone to soil homogeneity leading to the accumulation of more potential pathogens, such as *Fusarium*. In the heterogeneous soil of other crops, the relative abundance of pathogens decreases, which is better for plant growth [51]. The fungal communities of soil and plant roots seem to be more susceptible [52,53,54]. Current studies have shown that the main pathogens causing *L. barbarum* root rot are mostly fungi. It is reported that fungi and bacteria can also jointly induce disease [55], suggesting that the interactions between microorganisms are extremely complex. Nutrient input and tillage of the soil have been shown to spatially affect plants and microbes, which interact in countless ways in terrestrial ecosystems [56,57,58,59,60]. Probiotics can also “cooperate” with each other to maximize the acquisition of nutrients from the soil and promote growth [61]. The composition of microbial communities in the soil is crucial for maintaining soil function because they are involved in the formation of soil structure, and under natural conditions, mutual “feedback” between plants and soil usually affects soil microbial community composition [62,63]. For example, healthy soil can resist the invasion of pathogens in tobacco, especially pathogenic fungi, by regulating the structure of the soil microbial community. It can also inhibit the occurrence of soil-borne diseases by promoting the growth of beneficial bacteria [64]. Therefore, changes in the microbial community structure are very likely to destroy the diversity of microorganisms and cause various diseases. In addition, determining the impact of agricultural management practices, such as fertilization, tillage, crop rotation, etc., on root microbial communities is considered an integral part of sustainable agricultural research [65].

## 5. Conclusions

The richness of fungal communities in the rhizoplane of healthy *L. barbarum* plants was significantly higher than that in the rhizoplane of diseased plants. In healthy plants, the community evenness and diversity in the rhizoplane were significantly different than in the rhizosphere and root zone. The richness of the bacterial communities in the three parts of the healthy plants was greater than in the corresponding parts of the diseased plants. The dominant fungi and their proportions in the three parts (rhizoplane, rhizosphere, and root zone) of the healthy plants and the diseased plants were quite different. There was no significant difference in the composition of dominant bacteria in the three parts of the healthy plants and diseased plants at the genus level, but there were certain differences in the abundance. Functional prediction found that the abundance of functions belonging to metabolism in the bacterial community was the largest, and the functions of metabolism and genetic information processing of the diseased plants were lower. Fungal community function prediction showed that the Animal Pathogen-Endophyte-Lichen Parasite-Plant Pathogen-Soil Saprotroph-Wood Saprotroph group had the largest functional abundance, with the corresponding fungus being *Fusarium*.

## Figures and Tables

**Table 1:** Specific sample serial numbers.

	Rhizoplane	Rhizosphere	Root Zone
Diseased plants	QX_GBBZ (QX111, QX112, QX113)	QX_GJBZ (QX121, QX122, QX123)	QX_GWBZ (QX131, QX132, QX133)
Healthy plants	QX_GBJK (QX114, QX115, QX116)	QX_GJJK (QX124, QX125, QX126)	QX_GWBZ (QX134, QX135, QX136)

## Data Availability

Data are available upon request from the corresponding author.
